# Low-dimensional nanomaterials as an emerging platform for cancer diagnosis and therapy

**DOI:** 10.3389/fbioe.2023.1101673

**Published:** 2023-01-19

**Authors:** Fengzhi Cui, Jianhua Liu, Tianqi Zhang, Siwen Pang, Haijia Yu, Nannan Xu

**Affiliations:** Department of Radiology, The Second Hospital of Jilin University, Changchun, China

**Keywords:** 0D nanomaterials, 1D nanomaterials, 2D nanomaterials, imaging, cancer therapy

## Abstract

The burden of cancer is increasing, being widely recognized as one of the main reasons for deaths among humans. Despite the tremendous efforts that have been made worldwide to stem the progression and metastasis of cancer, morbidity and mortality in malignant tumors have been clearly rising and threatening human health. In recent years, nanomedicine has come to occupy an increasingly important position in precision oncotherapy, which improves the diagnosis, treatment, and long-term prognosis of cancer. In particular, LDNs with distinctive physicochemical capabilities have provided great potential for advanced biomedical applications, attributed to their large surface area, abundant surface binding sites, and good cellular permeation properties. In addition, LDNs can integrate CT/MR/US/PAI and PTT/PDT/CDT/NDDS into a multimodal theranostic nanoplatform, enabling targeted therapy and efficacy assessments for cancer. This review attempts to concisely summarize the classification and major properties of LDNs. Simultaneously, we particularly emphasize their applications in the imaging, diagnosis, and treatment of cancerous diseases.

## 1 Introduction

Cancer has been having a significant impact on public health concerns and it is emerging globally, being widely recognized as one of the main reasons for deaths among humans. Approximately 1,918,030 new cancer cases and 609,360 deaths cases were reported according to cancer statistics in 2022 and, despite global efforts, the incidence and mortality rates have been universally estimated to be increasing in recent years (Mullard, 2020; Siegel et al., 2022). Traditional anti-cancer treatment approaches are in use for cancer therapy today, usually composed of four categories, namely, surgical resection (Akinoso-Imran et al., 2022), radiotherapy (Duemer, 2016), chemotherapy (Godishala et al., 2018), and immunotherapy (Bergman, 2019), which have achieved a certain degree of success in relation to inhibiting cancer cells’ proliferation and lengthening patients’ survival periods. Due to the complexity, diversity, and heterogeneity in tumor cells, combined with the long treatment cycle, obvious side effects, high recurrence rate, and multidrug resistance (MDR) in tumor treatment (Pe’er et al., 2021; Zhang et al., 2022), traditional therapeutic methods are not obviously effective, with high rates of recurrence and metastasis (Peitzsch et al., 2017) that do not lead to a relatively favorable survival prognosis. Therefore, finding effective anti-cancer approaches for accurate diagnosis and treatment has extremely important application prospects.

Alongside the rapid progress of advanced nanotechnology, there exist many works applying nanomaterials in a wide variety of industries, leading to great advantages in particular for biomedical applications. LDNs have gained intense interest and attention for a variety of biomedical applications owing to their unique physicochemical characteristics, such as the large surface area, abundant surface binding sites, and good cellular permeation properties. Compared to conventional 3D nanomaterials, LDNs can be easily realized for effective drug-loading, exclusive surface modification, and functionalization, as well as targeted drug delivery, which provides valuable advanced multi-diagnostic approaches for efficient and precise tumor-targeted therapy (Gao et al., 2017; Zhou et al., 2022).

**FIGURE 1:**
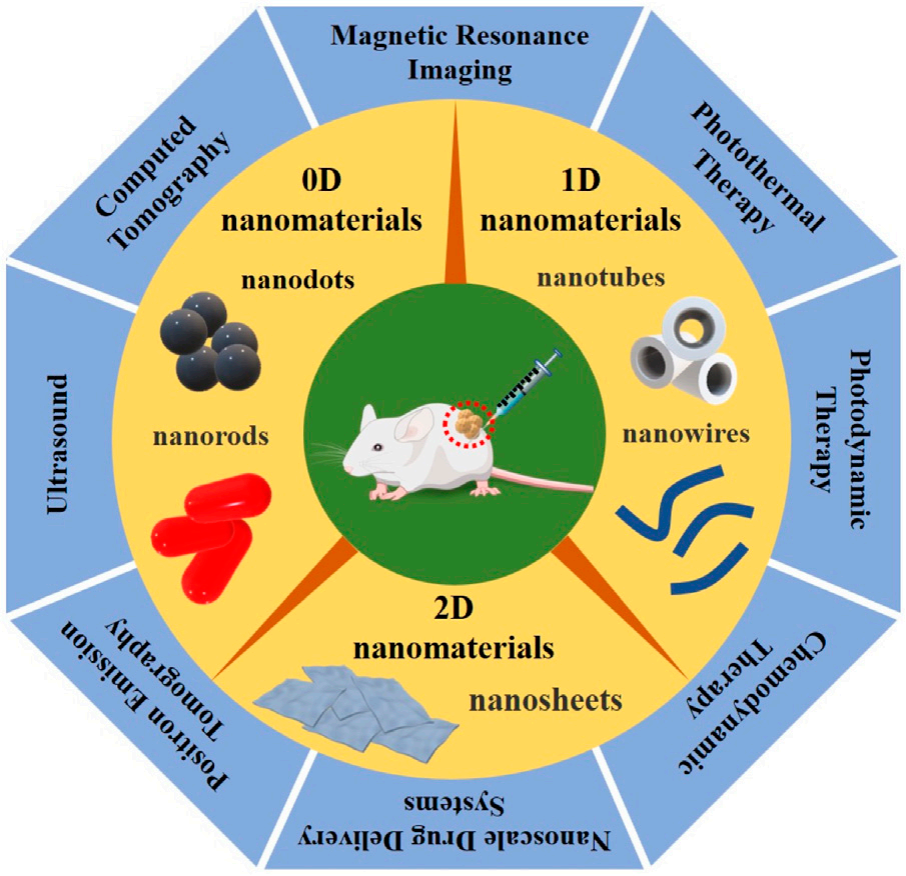
Graphical summarization of the three types of LDNs, according to dimensional and structure characteristics, divided into 0D nanomaterials (such as quantum dots and nanorods), 1D nanomaterials (such as nanowires and nanotubes), and 2D nanomaterials (such as nanosheets), combined with the novel therapeutic methods and imaging techniques, including PTT/PDT/CDT/NDDS and MRI/US/PAI/CT/PET, which have great promise for imaging-guided non-invasive cancer therapy.

LDNs combined with novel therapeutic methods, including PTT (photothermal therapy), PDT (photodynamic therapy), CDT (chemodynamic therapy), and NDDS (nanoscale drug delivery systems), have been shown to have great potential for non-invasive tumor therapy, which decreases the severity of surrounding normal histiocytic destruction. Tracking the biodistribution of nanoparticles in living tissues can be imaged with magnetic resonance (MR), ultrasound (US), photoacoustic (PA), computed tomography (CT), positron emission tomography (PET), and single-photon emission computed tomography (SPECT). Simultaneously, medical imaging modalities can also monitor the treatment procedure in real-time and detect the LDNs accumulating at the tumor site, achieving precise tumor localization and assessing the therapeutic effects, as seen in Figure 1. Thus, LDNs combined with novel therapeutic methods and imaging techniques have given birth to “nanomedicine.” Nanomedicine, as an emerging and rapidly expanding field, has become the most important field of medical research, constantly promoting medical advances (Kim, 2019; Yang et al., 2019; Lammers and Ferrari, 2020). Compared with traditional treatment methods, nanomedicine has significantly reduced tumor recurrence and metastasis, showing many advantages and great prospects concerning the optimization of therapeutic efficiency.

In this review article, we present a brief account of the classification and major properties of LDNs. In addition, we particularly emphasize their applications in the diagnosis and treatment of cancerous diseases.

## 2 Novel therapeutic methods for cancer

Unfortunately, eradicating tumors *via* monotherapy has been limited by its inherent defect, which is the lack of feasibility. An increasing number of recent studies have discovered that combination therapy can significantly improve patients’ symptoms and quality of life, which suggests a new therapeutic approach for cancer. In combination therapy, monotherapy can compensate for some disadvantages of other therapies. Therefore, the synergistic effect may not only improve the prognosis but also enhance the therapeutic effect of monotherapy. Currently, combined therapy is a clinical treatment recommended for its efficiency, and many potentially lucrative forms of combination therapy are being discovered.

### 2.1 Photothermal therapy for cancer

In addition to having limited side effects and being a high-efficiency cancer treatment, photothermal therapy (PTT) has the advantage of minimizing the risk of damage to normal tissues (Doughty et al., 2019). Photothermal agents (PTA) are the principal element in photothermal absorption, which plays an important role in PTT (Hwang and Jung, 2021). The basic principle of PTT is to use PTA to generate enough heat under the irradiation of near-infrared (NIR) and other external light sources to destroy and eliminate cancer cells, in which the strong light absorption and high photothermal conversion efficiency of PTA are the keys to the success of PTT (Nasseri et al., 2022). When the cancer tissue temperature reach to 42°C, this leads to local tissue irreversible damage, and with the increase of the reaction temperature, the following reactions will occur: protein denaturation; DNA synthesis weakening; oxygen-depletion; and pH levels dropping (Yu et al., 2020). Eventually, PTT effectively inhibits tumor recurrence and metastasis. In conclusion, compared with the traditional treatment of tumors, PTT has become one of the most promising and effective cancer treatments.

### 2.2 Photodynamic therapy for cancer

Photodynamic therapy (PDT) is a therapeutic modality for the local treatment of disease that requires the eradication of pathological cells based on the interaction of light, a photosensitizer (PS), and oxygen (Abrahamse et al., 2017). PDT treatment consists of three steps: first, the controlled PS forms selective endocytosis and retention in tumor cells; second, the PS is excited by light of the appropriate wavelength to release ROS, which includes singlet oxygen (^1^O_2_), peroxide (O_2_^2−^), superoxide (O^2−^), and hydroxyl radical (•OH), leading to the apoptosis, necrosis, and autophagy in cancer cells; third, PDT motivates a number of immune responses to cause inflammatory cells (Wang et al., 2021a). The PDT process destroys tumors through a variety of mechanisms, including the apoptosis and necrosis of neoplastic cells, the degeneration or shut-down of tumor blood supply, the stimulation of anti-tumor immune responses, and the induction of inflammation in the treated location. PDT has been regarded as an alternative therapy option, having remarkable therapeutic effects, leading to it being applied in cancers of various types and locations of therapy (Mansoori et al., 2019).

### 2.3 Chemodynamic therapy for cancer

Chemodynamic therapy (CDT) has emerged as a cutting-edge and effective cancer treatment method that can produce highly harmful hydroxyl radicals (•OH) from endogenous hydrogen peroxide (H_2_O_2_) under the presence of catalysts *via* a Fenton or Fenton-like reaction utilizing the unique feature of tumor microenvironments to eradicate cancer cells that can cause DNA necrosis, protein inactivation, lipid oxidation, and finally induce cell apoptosis or necrosis (Wang et al., 2020; Yang and Liu, 2020). In addition, Fenton and Fenton-like reactions can also produce O_2_, which can relieve the hypoxia and antioxidant capability of the tumor (Li et al., 2021). Furthermore, with a huge influx of “All-in-One” theranostic nano-agents, it is usually combined with chemotherapy, radiotherapy, phototherapy, sonodynamic therapy, and immunotherapy, which can not only improve the comprehensive synergistic therapeutic effect of cancer, but also enhance the anti-cancer therapeutic effect. In short, CDT presents a new route of treatment for highly efficient cancer theranostics.

### 2.4 Nanoscale drug delivery systems for cancer

One of the most important and significant potential opportunities in targeted drug delivery treatment modalities and diagnostic agents is nanoscale drug delivery systems (NDDS). In recent years, numerous chemotherapeutic drugs, inhibitors, vaccinations, proteins, and contrast agents have all been introduced. NDDS may be categorized into pH-sensitive delivery systems, enzyme-sensitive delivery systems, thermo-sensitive delivery systems, redox-sensitive delivery systems, and light-sensitive delivery systems, based on the unique features of the diseased microenvironment (Dheer et al., 2019; Li et al., 2019; Mirhadi et al., 2020). In addition, a variety of NDDS formulations, such as liposomes, nanoparticles, micelles, etc., have been designed as innovative cancer therapies. Especially in the area of tumor-targeted therapy, NDDS can not only resolve MDR and the recurrence of malignant tumors but also modify the immune response to improve the treatment of MDR cancer, providing more inspiration and thoughts for the precise treatment of tumors in the next stage (Wei et al., 2016).

## 3 Applications of LDNs

In accordance with their dimensional and structure characteristics, nanomaterials can be divided into 0D nanomaterials (such as quantum dots and nanorods) (Li Volsi et al., 2018), 1D nanomaterials (such as nanowires and nanotubes) (Cha et al., 2016), 2D nanomaterials (such as nanosheets) (Wang et al., 2021b), and 3D nanomaterials (such as nanocubes), in which the first three are collectively known as low-dimensional nanomaterials (LDNs) (Koh, 2010; Chai et al., 2021).

### 3.1 0D nanomaterials imaging diagnosis and treatment

Nanodots, ranging in size from 2 to 20 nm, are among the most representative LDNs, due to the characteristics of their chemical or physical properties, such as the low cost of synthesis, small size, low toxicity, excellent biocompatibility, chemical stability, and some attractive newly emerging properties, leading to them being at the forefront of cancer diagnostic imaging (Zou et al., 2017; Jia et al., 2018).

Lei et al. (2017) successfully synthesized ultrasmall poly-protected bismuth nanodots *via* an ultrafine strategy (Table 1). Due to their significant near-infrared absorption, nanodots exhibit outstanding photothermal conversion efficiency and can be used as a nano theranostic agent for PTT. Additionally, PVP-Bi nanodots demonstrate photothermal imaging and computed tomography (CT) imaging characteristics, which might successfully promote the PTT process. Additionally, PVP-Bi nanodots have high biocompatibility and low cytotoxicity in *in vitro* and *in vivo* experiments, indicating their excellent reliability for cancer imaging and treatment (Lei et al., 2017). In this direction, Li et al. (2017a) investigated ultrasmall PEG–Bi_2_S_3_ nanodots prepared according to a “hot injection” method (Table 1). Due to the presence of the Bi element with a larger X-ray attenuation coefficient, PEG–Bi_2_S_3_ nanodots possess the capacity for CT imaging. Additionally, Bi_2_S_3_ is a semiconductor with a low energy gap that exhibits significant NIR absorption. As a result, Bi_2_S_3_ might perform as a highly productive CT/PTT dual-functional agent by itself for imaging-guided photothermal therapy for effective and reliable cancer treatment (Li et al., 2017a). In their study, Xu et al. (2018) suggested that monodisperse Gd/Ru@BSA nanodots could be successfully synthesized through a simple protocol with high photothermal conversion efficiency, which achieved a significant efficacy for anti-cancer therapy under PTT both *in vitro* and *in vivo* (Table 1). Meanwhile, Gd/Ru@BSA nanodots exhibit superior T1-weighted magnetic resonance (MR) imaging ability to evaluate the treatment effect (Xu et al., 2018). Furthermore, Xu et al. (2019) found that phthalocyanine-based nanodots (ZnPc-NDs) have dual synergistic photodynamic/photothermal effects to realize the diagnostic function, demonstrated by HeLa tumor both *in vitro* and *in vivo* (Table 1). Gd(III) on the surface of nanodots provided magnetic resonance performances, having achieved magnetic resonance imaging-guided tumor phototherapy. The as-prepared nanodots, regarded as powerful and safe nano theranostic agents, have great clinical promise for cancer nano-imaging-guided theranostic nanoplatforms (Xu et al., 2019).

**TABLE 1:** Illustrative examples of LDNs and their biomedical applications.

LDNs	Type	Imaging	Therapy	Application	Reference
Poly-protected bismuth nanodots	0D nanomaterials	CT imaging	PTT therapy	Tumor tissue	Lei et al. (2017)
PEG–Bi_2_S_3_ nanodots	0D nanomaterials	CT imaging	PTT therapy	Tumor tissue	Li et al. (2017a)
Gd/Ru@BSA nanodots	0D nanomaterials	T1-weighted MRI	PTT therapy	Tumor tissue	Xu et al. (2018)
Phthalocyanine-based nanodots (ZnPc-NDs)	0D nanomaterials	T1-weighted MRI	PTT/PDT therapy	Tumor tissue	Xu et al. (2019)
Ag/BaGdF_5_: Yb^3+^, Er^3+^ nanocomposites	1D nanomaterials	CT/MR imaging	PTT therapy	Tumor tissue	Chen et al. (2019)
Si–Au	1D nanomaterials	PA imaging	PTT therapy	Tumor tissue	Sun et al. (2019)
Synthesized silicon nanowires (SiNWs)	1D nanomaterials		NDDS therapy	Tumor tissue	Peng et al. (2014)
Pd@Au, Pd@Ag nanoplates, and mesocrystalline Pd nanocorolla	2D nanomaterials	PA/CT imaging	PTT/chemotherapy; PTT/PDT therapy	Tumor tissue	Chen et al. (2017)
Nanosheets (MnO_2_ NSs)	2D nanomaterials	T1/T2-weighted MRI		Tumor tissue	Zhao et al. (2014)
TiNSs-PEG nanosheets	2D nanomaterials	PA and CT imaging	PTT therapy	Tumor tissue	Xie et al. (2019)
MnO_2_ nanosheets (M-NSs)	2D nanomaterials	PA and PET imaging	PTT therapy	Tumor tissue	Tang et al. (2019)
MUCNPs@BPNs-Ce6	2D nanomaterials	T1/T2-weighted MR and US imaging	PTT/PDT therapy	Tumor tissue	Zhang et al. (2020)

### 3.2 1D nanomaterials imaging diagnosis and treatment

One-dimensional (1D) nanostructures with characteristic surface-to-volume and aspect ratios, such as nanowires (NWs), nanobelts (NBs), nanoneedles (NNs), nanorods (NRs), nanotubes (NTs) (Contreras-Torres et al., 2022), and nanofibers (NFs), which might adsorb or conjugate to various therapeutic compounds, have a significant benefit in terms of disease diagnostics, monitoring, and efficacy evaluation (Roehlecke and Schmidt, 2020).

Chen et al. (2019) discovered that Ag/BaGdF_5_: Yb^3+^, Er^3+^ nanocomposites, synthesized by Ag nanowires and PVP-modified BaGdF_5_: Yb^3+^, Er^3+^ spherical nanoparticles *via* convenient solvothermal and hydrothermal procedure, exhibit remarkable performance on photothermal conversion and CT/MR imaging efficiency (Table 1). Further evidence of the PTT effect was validated by Hela cells under a 808 nm laser. Meanwhile, CT/MR was used for the assessment of cancer therapeutic effects. Experimental results showed that the obtained nanoparticles not only possess an extraordinary photothermal conversion effect but also an excellent imaging capability for CT/MR (Chen et al., 2019). In this direction, Sun et al. (2019) investigated silicon nanowires decorated with gold nanoparticles (Si–Au) that were synthesized *via* a simple and convenient method (Table 1). Si–Au showed a good photothermal effect under NIR-I in *in vitro* and *in vivo* experiments, and it also demonstrated highly efficient PA imaging under NIR-II in 4T1 to guide photothermal cancer therapy (Sun et al., 2019). Due to the abundant surface binding sites in 1D nanomaterials, they can conjugate to various therapeutic molecules. In this direction, Peng et al. (2014) synthesized silicon nanowires (SiNWs) of large-area porous structures with high drug-loading capacity through a facile route and loaded doxorubicin (DOX) to remove drug-resistant cancer cells (Table 1). *In vitro* experiments showed that nanoparticles with a low RF value are extremely effective for reversing drug resistance. SiNWs-DOX solves the question of cancer resistance to the drugs and prevents blindly using the medicine (Peng et al., 2014). Additionally, as observed in some research, 1D nanomaterials have characteristic surface-to-volume and aspect ratios, making them potential carriers of biomolecules and chemotherapeutic medications for various cancer-related treatments (García-Hevia et al., 2016).

### 3.3 2D nanomaterials imaging diagnosis and treatment

Among nanomaterials, 2D nanomaterials (2D-NMs) have gained considerable interest from researchers due to their physical and chemical properties, including their planar structure, ultra-thin thickness, and ease of functionalization (Mohammadpour and Majidzadeh, 2020). Furthermore, 2D-NMs are capable of adsorbing or conjugating various contrast agents, enabling rapid multimodal imaging with a quick turnaround time, as well as the detection of tumors and metastases (Ashfaq et al., 2022).

Chen et al. (2017) summarized Pd@Au, Pd@Ag nanoplates, and mesocrystalline Pd nanocorolla applications in cancer diagnosis and therapy (Table 1). The different forms of 2D Pd nanosheets were used to carry out extensive research because of their many benefits. The main advantages are a large surface area, strong absorption in the NIR region, high photothermal conversion efficiency, high photothermal stability, and excellent PA or CT imaging capability. Experimental results indicated that the combination of PTT/chemotherapy and PTT/PDT therapy achieved high synergistic therapeutic effects, realizing PA and CT imaging-guided PTT for cancer treatment (Chen et al., 2017). Another successful implementation of manganese dioxide nanosheets (MnO_2_ NSs) is presented in the work of Zhao et al. (2014), where MnO_2_ NSs were employed for MRI bimodal tumor cell imaging (Table 1). Moreover, Xie et al. (2019) discovered that Ti nanosheets (NSs) exfoliated through simple methods (Table 1). The experiment confirmed that TiNSs-PEG possesses excellent light absorbance, preeminent photothermal performance, and stability. Therefore, TiNSs-PEG combined with PTT can achieve high tumor cell-killing efficiency. The effectiveness of treatment can be assessed using dual-modal contrast-enhanced PA and CT imaging (Xie et al., 2019). Tang et al. (2019) synthesized MnO_2_ nanosheets (M-NSs) for a tumor-targeted multimodal diagnostic imaging probe *via* a one-step wet-chemical method. Through the surface functionalization of the M-NSs, 64Cu radionuclides were successfully chelated onto nanoparticles to achieve PET imaging. In addition, the obtained nanomaterials not only possessed a good photothermal therapeutic effect but also realized effective photoacoustic-imaging-guided synergistic starvation-enhanced photothermal therapy (Tang et al., 2019). Zhang et al. (2020) designed MUCNPs@BPNs-Ce6 nanocomposites for tumor phototherapy guided by multimodal imaging. BPNs and Ce6, as a component of nanomaterials with high photothermal conversion efficiency, were employed to promote PTT and PDT under a single irradiation light of 808 nm. In addition, Fe_3_O_4_ and MnO_2_ nanoparticles realized T1/T2-weighted magnetic resonance imaging, and Ce6 has the capability of fluorescence imaging. It was proved by experiments that the bubbles produced by H_2_O_2_ decomposition in the tumor microenvironment could be used for ultrasound imaging. Further, MnO_2_ can transform excess H_2_O_2_ in the tumor microenvironment into O_2_ to enhance PDT. The obtained MUCNPs@BPNs-Ce6 not only enhances the therapeutic effect of PDT/PTT but also enables multimodal imaging to evaluate the therapeutic effect of cancer in real-time (Zhang et al., 2020). The development of effective diagnostic and cancer therapies relies on the development of 2D nanomaterials, which are constantly expanding. Therefore, the development of 2D-NMs will improve clinical decision-making and advance precision medicine (Li et al., 2017b; Wang et al., 2017; Zhao et al., 2022; Zhao et al., 2023).

## 4 Outlooks

In recent years, LDNs have emerged and attracted intense attention due to their exclusive physical and chemical properties, such as their small size, high durability, chemical stability, biocompatibility, non-toxicity, environmental friendliness, and the ease of chemical functionalization and surface modification. LDN-based nano-imaging-guided theranostic nanoplatforms have opened new frontiers in cancer precision medicine, integrated imaging and therapy into multimodal theranostic nanoplatforms to achieve individualized diagnostic accuracy and therapy for patients (Aminolroayaei et al., 2021; Huang et al., 2023). However, transforming academic research into clinical practice is still a challenging and currently unresolved problem in anti-cancer clinical practice therapies. In the context of the clinical effectiveness and safety of drugs, LDNs are expected to realize clinical application by continually improving nanomaterials’ performance in the future. In conclusion, theranostic nanoplatforms based on LDNs have the tremendous potential to be employed as cancer therapies and diagnostic tools, providing more powerful and useful means for biologists and clinicians (Cheng and Zhang, 2018; Li et al., 2022).
